# SpyTagged Mimotope Peptide Mediated Competitive Antigen-Based Rapid Quantitative Immunoassays for Uniconazole Residue

**DOI:** 10.3390/foods14244358

**Published:** 2025-12-18

**Authors:** Tailong Wei, Xiao Chen, Chong Cai, Yuanzhen Guo, Mengjun Zhou, Qiannan Gao, Qinghua He

**Affiliations:** 1 State Key Laboratory of Food Science and Resources, Nanchang University, No. 235 Nanjing East Road, Nanchang 330047, China; 357900210015@email.ncu.edu.cn (T.W.); 19326409190@163.com (X.C.); 357900240012@email.ncu.edu.cn (C.C.); 407900230007@email.ncu.edu.cn (Y.G.); 417900230134@email.ncu.edu.cn (Q.G.); 2 Jiangxi General Institute of Testing and Certification Instituto for Food Control, Nanchang 330052, China; 13767176735@163.com; 3 Sino-German Joint Research Institute, Nanchang University, No. 235 Nanjing East Road, Nanchang 330047, China; 4 In Vitro Diagnostic Technology Innovation Center for Nanobody, No. 1166 Yiyuan Road, Nanchang 330038, China

**Keywords:** UCZ, phage display, mimotope, LFIA, magnetic beads, self-assembly

## Abstract

Mimotope-based immunoassays offer an eco-friendly alternative to chemically synthesized antigens for the quantitative analysis of small molecules, but their use for practical on-site and high-throughput residue monitoring remains limited. Herein, we report the selection, production, and application of a phage display–derived mimotope targeting an anti-uniconazole monoclonal antibody (UCZ-mAb), with the aim of developing two complementary immunoassays that enable sensitive, eco-friendly detection of UCZ residues in agricultural samples. A 12-mer phage-displayed peptide library was screened to identify UCZ-specific mimotopes, and a selected sequence was genetically fused to SpyTag and expressed in *Escherichia coli* to generate a SpyTagged mimotope. Leveraging the SpyCatcher/SpyTag self-assembly system, the SpyTagged mimotope was directionally conjugated onto SpyCatcher-functionalized time-resolved fluorescence beads (TRFBs) and subsequently used as a signal-labeled competitive antigen in a lateral flow immunoassay (LFIA) designed for rapid on-site screening. In parallel, a wash-free magnetic separation immunoassay (MSIA) suitable for green, high-throughput screening in routine laboratories was established using self-assembled mimotope-TRFB probes. The LFIA and MSIA exhibited half-maximal inhibitory concentrations (IC_50_) of 3.70–6.72 μg/kg and 16.4–18.3 μg/kg, respectively, in real samples. Spiked-sample recoveries ranged from 91.1 to 107.8% for LFIA and 92.6–115.7% for MSIA, demonstrating acceptable accuracy and precision. These results indicate that the SpyTagged mimotope–based LFIA and MSIA provide complementary, reliable, and sensitive platforms for on-site screening and high-throughput monitoring of UCZ residues in agricultural samples, while avoiding the drawbacks associated with traditional chemical antigen synthesis.

## 1. Introduction

The development of analytical methods for small molecule compounds has important implications for a variety of fields, such as clinical diagnostics, drug discovery, environmental monitoring, and food analysis [1]. Sensitive, simple, and rapid analytical methods suitable for on-site monitoring by untrained users are essential and in high demand. Immunoassays, which are based on antigen–antibody specific recognition, have gained widespread application due to their high sensitivity and ease of use [2,3,4]. Immunoassays have been adapted to different tracer reagents and detection platforms resulting in a variety of immunoassay modes, such as enzyme-linked immunosorbent assay (ELISA) [5], lateral flow immunoassay (LFIA) [6], magnetic separation immunoassay (MSIA) [7], electrochemical immunosensors [8], and homogeneous assays [9,10,11]. For the analysis of small molecule compounds, the competitive immunoassay format, which relies on specific antibodies and antigens, is most commonly employed because small molecule compounds typically contain only a single epitope [12]. Traditionally, antigen preparation relies on chemical synthesis, in which small-molecule compounds are conjugated to carrier proteins for use as coating or detection reagents [13]. This approach presents several drawbacks, including complex synthetic procedures, poor homogeneity, and risks associated with toxic reagents that may endanger operator safety and the environment [14,15,16,17].

Therefore, eco-friendly and easy-to-produce substitutes for chemosynthetic antigens in immunoassays should be developed. Since the introduction of phage display technology, anti-idiotypic nanobodies and mimotope peptides have been utilized as substitutes for chemically synthesized antigens [18,19]. Mimotope peptides can also be readily engineered and offer a broad range of applications, as their clear gene sequences enable the analysis of structure-activity relationships with antibodies and facilitate rational mutations to enhance the sensitivity of immunoassays [20,21].

Due to their small molecular weight and susceptibility to degradation, mimotope peptides need to be fused to other proteins through genetic engineering techniques, such as maltose-binding protein (MBP), which is used as a coating antigen applied in ELISA and LFIA [16,22]. Mimotope peptides fused with yellow fluorescent protein (YFP) have also been developed as a homogenous immunoassay based on fluorescence quenching between antibody-coupled gold nanoparticles and mimotope peptide fused with YFP [23]. To further enhance the detection sensitivity of immunoassays in terms of signal intensity, fluorescent materials, such as quantum dot nanobeads (QBs) [24], time-resolved fluorescence beads (TRFBs) [25] and aggregation-induced emission (AIE) [26] have been employed in coupling with antibodies or antigens. However, antibodies and antigens are prone to reduced activity during coating or chemical conjugation processes for changes in structure [27]. The SpyCatcher/SpyTag system, developed from the CnaB2 domain of the fibronectin adhesion protein FbaB from *Streptococcus pyogenes*, is particularly promising for the gentle coupling reaction and stable isopeptide bond in bioorthogonal conjugation [28].

Uniconazole (UCZ) is a triazole-type plant growth regulator used for reducing stem elongation and improving lodging resistance and yield in cereals, fruits, and horticultural crops [29]. Although UCZ is intentionally applied in agriculture rather than being an incidental contaminant, its persistence can lead to residues in edible commodities, posing toxicological risks [29]. To date, UCZ detection has primarily relied on instrumental methods like liquid chromatography-mass spectrometry (LC-MS/MS), which are sensitive but costly and unsuitable for on-site screening [29]. In contrast, biosensors and immunoassays for UCZ are scarce, with limited reports on triazole regulators using ELISA or electrochemical approaches [30,31,32], highlighting the need for novel, eco-friendly immunoassay formats to enable rapid, high-throughput screening in complex food matrices and complement confirmatory analyses. Therefore, UCZ was selected as the research subject. A phage-displayed UCZ mimotope peptide that binds to the anti-UCZ monoclonal antibody (UCZ-mAb) was screened from a 12-mer phage-displayed peptide library. And the selected UCZ-mimotope peptide was fused with SpyTag to generate UCZ SpyTagged mimotope, which was directionally coupled to TRFB-SpyCatcher via self-assembly. We developed an LFIA based on SpyTagged mimotope. To further increase assay throughput and reduce processing time, the self-assembly probes were combined with immunomagnetic beads to establish a green, wash-free, and high-throughput magnetic separation immunoassay (MSIA) exhibiting a positive correlation signal, which meets institutionally specified limits of detection for UCZ residues in crops and vegetables.

## 2. Materials and Methods

### 2.1. Main Chemicals and Reagents

The Ph.D-12 phage display peptide library was purchased from New England Biolabs (Ipswich, CA, USA). The UCZ-mAb, UCZ-OVA [33], and SpyCatcher [34] were previously prepared by our lab. Time-resolved fluorescent beads (TRFBs) were purchased from Vdo Biotech (Suzhou, China). Carboxyl magnetic beads were purchased from Beaverbio (Suzhou, China). The standard of UCZ, paclobutrazol (PBZ), 2-methyl-4-chlorophenoxyacetic acid (MCPA), and N-Hydroxysulfosuccinimide sodium salt (NHSs) and carbodiimide hydrochloric acid (EDC) was supplied by Aladdin (Shanghai, China). The standard of zearalenone (ZEN), aflatoxin B_1_ (AFB_1_), deoxynivalenol (DON), fumonisins B_1_ (FB_1_) and ochratoxin A (OTA) were purchased from Sigma-Aldrich Chemical Co. (St. Louis, MO, USA). Anti-His mAb was purchased from Jiangsu Cowin Biotech Co. (Taizhou, China). The material consisting of the strip test (the sample pad, the absorbent pad and the conjugate pad) was purchased by Jie Yi, Ltd. (Shanghai, China). Unless otherwise stated, all reagents are of analytical grade.

### 2.2. Biopanning and Identification of Phage Displayed Mimotope Peptide for UCZ

**Scheme 1:**
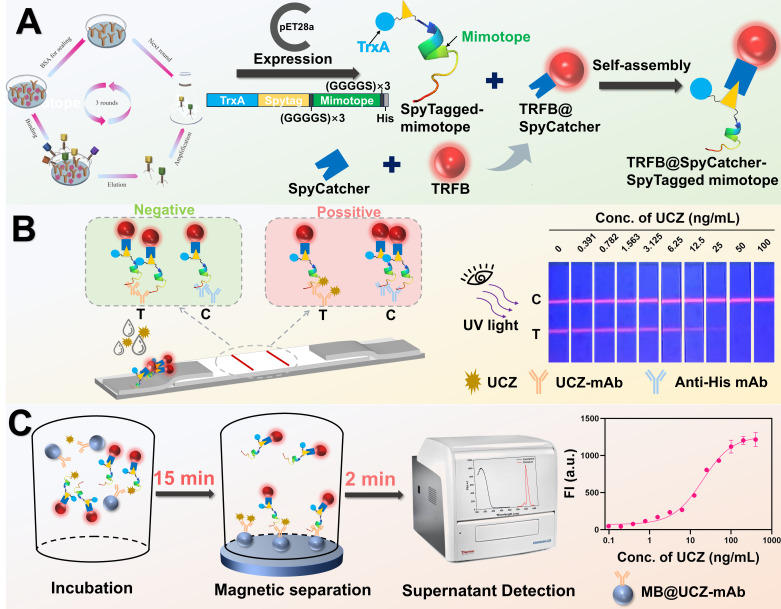
Schematic of the SpyTagged mimotope-based immunoassays for UCZ. (**A**) Preparation of self-assembly probes based on SpyTagged mimotope. (**B**) SpyTagged mimotope-based LFIA for UCZ detection. (**C**) SpyTagged mimotope-based MSIA for UCZ.

The process for biopanning phage displayed mimotope peptide for UCZ was shown in Scheme 1A. The Ph.D-12 phage display peptide library was subjected to three rounds of panning using UCZ-mAb as the target with a method described previously. In the first round of panning, microplate wells were coated with 100 μg/mL of UCZ-mAb overnight at 4 °C, and after 3% BSA blocking, 2.0 × 10^11^ pfu Ph.D-12 phage library was incubated in wells. The unbound phages were washed via 0.1% PBST, bound phages were eluted with 0.1 M Gly-HCl (pH 2.2), neutralized using 1 M Tris-HCl (pH 8.5), and amplified for subsequent rounds. The next two rounds of phage biopanning were performed similarly to the first round, and the details are seen in Table S1. The third round of eluted phages was collected, and an individual phage isolate from the elution was evaluated for UCZ-mAb binding by phage ELISA and DNA sequencing [19,35].

To gain a comprehensive understanding of the recognition mechanism between UCZ-mAb and UCZ mimotope, a series of post-sequencing analyses were conducted, including homology modeling and molecular docking. The molecular docking results of UCZ-mAb and UCZ mimotope were performed via Alphfold3 and presented in the form of an interaction diagram created with Ligplot^+^ V.2.2.

### 2.3. Construction Plasmid for SpyTagged Mimotope for UCZ

In order to biosynthesize UCZ mimotope peptide, we adopted a strategy of tandem fusion expression by linking the peptide, a solubility-enhancing tag protein (TrxA), and SpyTag to construct SpyTagged mimotope with self-assembly capability, as shown in Scheme 1A. The N-terminus of the UCZ mimotope gene was linked with the SpyTag gene through a flexible linker (GGGGSGGGGSGGGGS), C-terminus was linked with 6xHis for purification and inserted into pET32a (contains *Trx A* gene) for expression.

### 2.4. Expression and Characterization of SpyTagged Mimotope

Expression. The constructed expression plasmid was transformed into *E. coli* BL21 cells to express the SpyTagged mimotope. In the exponential phase of *E. coli* BL21 cells, the final concentration of 0.2 mM IPTG was added to cultures and induced expression at 25 °C for 12 h. After centrifugation, the culture supernatant was broken to expose the fusion protein under an ultrasonic crusher. Then, the fusion proteins were purified with Ni resin and dialyzed with PBS. The purification was verified by sodium dodecyl sulfate-polyacrylamide gel electrophoresis (SDS-PAGE) on 12% gels.

Self-assembly and characterization. The purified 100 μM SpyTagged mimotope and 100 μM SpyCatcher were mixed in equal volumes and incubated at 25 °C. The binding effect of incubation at 5–240 min was identified by SDS-PAGE on 12% gels. The performance of SpyTagged mimotope and assembled with SpyCatcher was identified by indirect competitive ELISA (ic-ELISA).

### 2.5. Preparation of Self-Assembly Probes Based on SpyTagged Mimotope

Preparation of the TRFB-SpyCatcher. On the basis of the SpyTagged mimotope peptide, self-assembly probes based on SpyTag/SpyCatcher were designed: the SpyCatcher was coupled with TRFBs to prepare TRFB-SpyCatcher (TRFB@SC), and then the SpyTagged mimotope was connected through self-assembly to obtain TRFB@SC-SpyTagged mimotope (TRFB@SC-STM). The TRFB@SC was prepared through the formation of an amide bond between the carboxyl group of TRFBs and the amino group of SpyCatcher using the EDC/NHSs method [36]. Specifically, 50 μg of TRFBs were coupled with 20 μg of SpyCatcher and then blocked by 0.2% Casein Na salt.

Self-assembly of TRFB@SC and SpyTagged mimotope. The TRFB@SC was mixed with 10 μg SpyTagged mimotope and rotated for 2 h at 25 °C. The mixture was then centrifuged at 9000 rpm for 10 min, and the sediment was collected and resuspended in 200 μL of reconstitution solution (25 mM Tris + 150 mM NaCl + 0.05% Tween-20 + 1% BSA + 5% trehalose + ddH_2_O). The TRFB@SC-STM was stored at 4 °C for future use.

### 2.6. SpyTagged Mimotope-Based LFIA for UCZ Detection

Assembly of LFIAs. The test strips were assembled using the following parameters: A nitrocellulose (NC) membrane (25 mm × 300 mm) was immobilized onto a polyvinyl chloride (PVC) backing plate (60 mm × 300 mm). Subsequently, an absorbent pad (17 mm × 300 mm), a conjugate pad (6 mm × 300 mm), and a sample pad (17 mm × 300 mm) were sequentially laminated onto the PVC substrate with 2 mm overlapping between adjacent components. UCZ-mAb and anti-His mAb were precisely dispensed onto the NC membrane using a BioDot XYZ3060 Platform (GHS510) dispensing system. Specifically, UCZ-mAb (0.8 mg/mL) and anti-His mAb (0.5 mg/mL) were sprayed on the detection line (T-line) and the quality control line (C-line), respectively. Afterward, the strips were dried at 37 °C for 2 h and cut to a width of 4 mm using a guillotine cutter(Autokun, Hangzhou, China). The assembled test strips were used for subsequent testing experiments.

Optimization of LFIA. Optimization of probes is the key to improving the analysis performance. For the LFIA, we optimize the preparation parameters of TRFB@SC-STM. The buffer pH (6.0, 6.5, 7.0, 7.5, 8.0, and 8.5), the amount of SpyCatcher (2, 5, 10, 15, 20, and 25 μg), and the incubation time of SpyTagged mimotope coupled with TRFB@SC (0.5, 1.0, 1.5, 2.0, 2.5, and 3 h). Subsequently, the amount of UCZ-mAb on T line, the amount of SpyTagged mimotope, and the amount of TRFB@SC-STM were optimized by orthogonal experiment. Finally, the pH of buffer (6.0, 6.5, 7.0, 7.5, 8.0, and 8.5) and concentration of methanol (5, 10, 20, and 25%) were analyzed to determine the optimal conditions. Unless otherwise stated, all experiments were performed in three independent replicates (*n* = 3).

Detection performance. To measure the detection sensitivity of UCZ in LFIA, different concentrations of standard UCZ solutions were diluted with PBS (10 mM, pH 7.4) to final concentrations of 0–100 ng/mL. 2 μL of TRFB probes and 80 μL of standard UCZ solutions were premixed and then dropped onto the sample pad. After 15 min, the fluorescence intensity (FI) of T and C lines was recorded by a portable fluorescent strip reader. The competitive inhibition curves of LFIA for UCZ quantification were built by plotting B/B_0_ against the logarithm of UCZ concentration, where B and B_0_ stand for the T/C ratios obtained from UCZ-positive and UCZ-negative samples.

### 2.7. SpyTagged Mimotope-Based MSIA for UCZ

Preparation of Immunomagnetic Beads. The immunomagnetic beads were prepared via coupling with magnetic beads and UCZ-mAb, similar to TRFB@SC. Briefly, 500 μg of MBs were dissolved in 1 mL of PB (0.01 M, pH 6.0) and dispersed by ultrasonic dispersion for 5 min. Next, 4 μL of EDC (10 mg/mL) and 8 μL of NHSs (10 mg/mL) were added to the dispersed solution of MBs, after ultrasonic dispersion, and then incubated for 30 min at room temperature under gentle rotation. After magnetic separation, the precipitate was resuspended with 0.45 mL of 0.01 M PB (pH 7.0) and mixed with 20 μg of UCZ-mAb for 2 h. After blocked by Casein Na salt and then magnetic separation, resuspended in 500 μL of reconstitution solution.

Optimization. To develop a MSIA for UCZ detection based on SpyTagged mimotope, the synthesis parameters of immunomagnetic beads and the detection conditions were optimized: the volume of MB@mAb (0.39, 0.78, 1.56, 3.13, 6.25, 12.5, 25 μL) in detection system and incubation time of detection system (3, 5, 8, 10, 12, 15, 18 and 25 min).

Detection performance. As shown in Scheme 1C, the MSIA of specific steps is as follows: 6.25 μL of MB@mAb and 1 μL of TRFB@SC-STM are diluted with 100 μL of PBS, respectively, and mixed per sample. Mix 100 μL of the mixture and 100 μL of UCZ standard (0–400 ng/mL) in 10% methanol-PBS into a black 96-well plate. After incubation for 15 min at 37 °C, followed by magnetic separation for 2 min, 100 μL of supernatant was collected and measured by a multifunctional enzyme immunoassay analyzer (EX: 340 nm, EM: 613 nm). The limit of detection and quantification for the assays were calculated by 10–maximal inhibitory concentrations (IC_10_) and 20–maximal inhibitory concentrations (IC_20_), respectively, obtained from four-parameter logistic curve fits formula:

(1) $$y=A_{2}+(A_{1}-A_{2})/\left(1+{(x/x_{0})}^{p}\right)$$

where *A*_1_, *A*_2_ are the fitted top and bottom of the curve, *p* denotes the hillslope of the curve, while *x*_0_ equals half-maximal inhibitory concentrations (IC_50_). where *A*_1_, *A*_2_ are the fitted top and bottom of the curve, *p* denotes the hillslope of the curve while *x*_0_ equals half-maximal inhibitory concentrations (IC_50_).

### 2.8. Performance Evaluation of LFIA and MSIA Based on SpyTagged Mimotope

To minimize interference from the real sample matrix, the samples (wheat, brown rice, and cabbage) were pretreated with Quick, Easy, Cheap, Effective, Rugged, Safe (QuEChERS) methods [37]. Briefly, 5 g of homogenized samples, 5 mL of 1% acetic acid-acetonitrile, 2 g of MgSO_4_, and 1 g of anhydrous sodium acetate were added to a 50 mL plastic centrifuge tube, shaken vigorously for 2 min, and then centrifuged at 4000 rpm for 5 min. After this, 2 mL of supernatant was removed and 1 g of MgSO_4_, 20 mg of graphitized carbon black and 40 mg of N-propylethylenediamine were added, and centrifuged at 4000 rpm for 5 min. 1 mL of supernatant was removed, filtered through a 0.22 µm nylon membrane and nitrogen-blown until nearly dry [38]. Finally, the dried samples were resuspended in 1 mL of 10% methanol-PBS to obtain the sample extracts. The UCZ at 20, 50 and 100 μg/kg in spiked samples, respectively, was detected to calculate the recovery by MSIA and LC-MS/MS. Each experiment was conducted in triplicate. The mycotoxins and pesticides do not share the same group as UCZ, and were included as typical co-contaminants that may occur in cereal-based matrices such as brown rice and wheat, in order to check possible non-specific interference from other common toxins extracted together with UCZ. PBZ has the same triazole group as UCZ and was used for specificity.

## 3. Results and Discussion

### 3.1. Selection and Identification of UCZ Mimotopes from Phage Display Peptide Library

**Figure 1:**
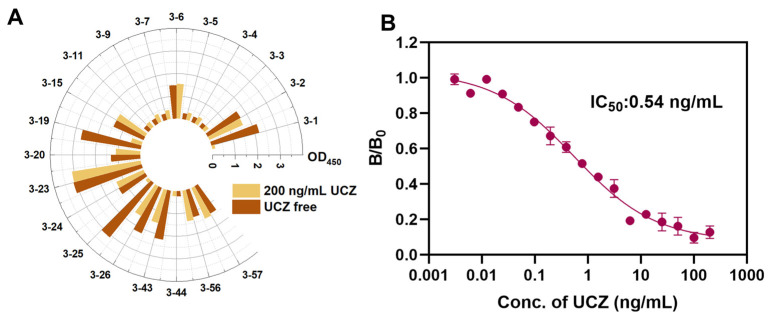
(**A**) Identification of UCZ mimotope from phage display peptide library. (**B**) Competitive inhibition curves of phage ic-ELISA.

To obtain mimotopes of UCZ, three rounds of panning were carried out with increasing selection pressure. The phage output from the first round to the third round was 5 × 10^5^, 3.4 × 10^6^ and 2.8 × 10^6^ pfu/mL, respectively. Among the 63 clones picked from the third-round titration plate, 22 clones tested positive in the ELISA (P/N > 2.1), yielding a positivity rate of 34.9%. As shown in Figure 1A, a total of 3 individual phages with the best signal-to-background ratios were successfully assayed in ELISA. After DNA sequencing and analysis, only one peptide sequence was identified from these 3 positive phage clones, renamed as 3-1 (I-P-F-K-E-G-L-G-F-I-S). To evaluate the analytical performance of the 3-1, competitive inhibition curves and of phage ELISA were plotted. As shown in Figure 1B, the IC_50_ of 3-1 was 0.54 ng/mL.

### 3.2. Analysis of Interaction Between UCZ-mAb and UCZ Mimotope Peptide

**Figure 2:**
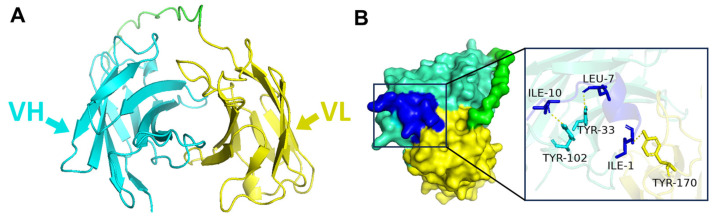
(**A**) The structure of UCZ-mAb, Cyan: Heavy chain, Yellow: Light chain. (**B**) Binding sites and interactions between UCZ-mAb and UCZ mimotope peptide.

The molecular docking between UCZ-mAb and UCZ mimotope 3-1 was essential to rationalize the mimotope’s binding efficacy and guide subsequent assay development, as structural insights can explain selectivity and affinity in competitive immunoassays [20,21]. Firstly, the UCZ-mAb and UCZ mimotope 3-1 were constructed using Alphfold3, which demonstrated high credibility as illustrated in Figure 2A,B. The scFv, comprising antiparallel β-sheets and typical heavy-chain variable (VH) and light-chain variable (LH) domains in green and yellow, is illustrated in Figure 2A. The molecular docking results of UCZ-mAb and UCZ mimotope 3-1 were performed via Alphfold3 and presented in the form of an interaction diagram created with Ligplot^+^ V.2.2. As illustrated in Figure 2B, the amino acids ILE-10, LEU-7 and ILE-1 in the UCZ mimotope 3-1 and TYR-33, TYR-102, TYR-170 in the CDR region of UCZ-mAb engage in hydrogen bond interactions to stabilize intermolecular interactions. These amino acids may represent the key sites for the binding of UCZ-mAb to UCZ mimotope 3-1, likely driven by hydrophobic and hydrogen bonding forces that mimic the triazole ring and side chains of UCZ. This interaction profile is consistent with mechanisms observed in other mimotope-antibody complexes for small-molecule pesticides, where tyrosine residues in CDRs facilitate stable binding and contribute to high specificity.

### 3.3. Characterization of SpyTagged Mimotope

**Figure 3:**
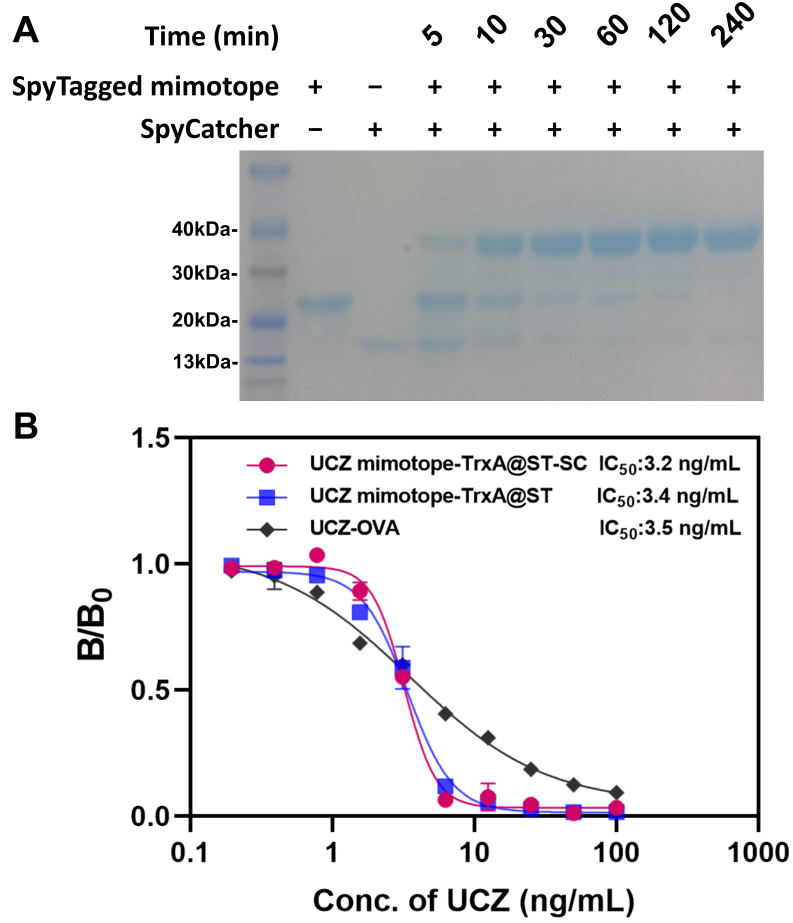
Self-assembly and characterization of UCZ SpyTagged mimotope and SpyCatcher. (**A**) SDS-PAGE (“+” indicates presence, “—” indicates absence). (**B**) ic-ELISA based on UCZ-OVA. UCZ SpyTagged mimotope and UCZ SpyTagged mimotope-SpyCatcher.

Purification and self-assembly of the SpyTagged mimotope were analyzed by SDS-PAGE. As shown in Figure 3A, the SpyTagged mimotope and SpyCatcher have only one band, suggesting high purity. Under the conditions of a SpyTagged mimotope/SpyCatcher molar ratio of 1:1 and 25 °C for 2 h, the coupled reaction is essentially complete. These results show that SpyTagged mimotope can be coupled with SpyCatcher conveniently and efficiently at 25 °C, attributed to the spontaneous isopeptide bond formation in the Spy system, which provides high yield and stability [28]. Subsequently, the performance of SpyTagged mimotope, SpyTagged mimotope-SpyCatcher, and UCZ-OVA was identified via ic-ELISA; their IC_50_ were 3.2, 3.4 and 3.5 ng/mL, respectively (Figure 3B). This indicates that biological coupling via SpyTag/SpyCatcher has no adverse effect on mimotope activity, likely because it preserves the peptide’s conformation and orientation. Moreover, the sensitivity is similar to chemically synthesized antigens, supporting the rationale for this eco-friendly approach in immunoassay development [18,19].

### 3.4. Characterization of TRFB@SC-STM

The characterization results are illustrated in Figure S1. As shown in Figure S1A,B, the hydrodynamic diameters (*D*_H_) of TRFB, TRFB@SC, and TRFB@SC-STM decreased sequentially; compared with TRFB (−19.3 mV), TRFB@SC showed a considerable change in charge (−22.4 mV) after the coupling with SpyCatcher, which confirmed the preparation of TRFB@SC. The zeta potential of TRFB@SC-STM (−24.3 mV) further deceased that indicated the successful self-assembly of SpyTagged mimotope and TRFB@SC. The TEM result of the TRFBs was presented as uniform and monodisperse sizes of 300 nm (Figure S1C). The assessment of the effects of modification on the Ex and Em wavelengths of TRFB@SC-STM showed that the modification had negligible effects on the properties of the TRFBs (Figure S1D).

### 3.5. Optimization of TRFB@SC-STM

To improve the performance of TRFB@SC-STM in immunoassays, TRFB@SC-STM was optimized on the LFIA platform. As shown in Figure S2A, the fluorescence intensity of the control line was measured after the probes were tested on the strip. pH 8.0 was determined as the optimal pH value in coupling for the highest FI of T-line, indicating that the TRFB@SC is stable at pH 8.0. The results in Figure S2B indicated that TRFB@SC obtained the maximum FI of T-line in the amount of 15 μg SpyCatcher per 30 μg TRFBs. The incubation time of self-assembly between TRFB@SC and STM, determined on the highest FI of T-line and inhibition rate, was 2 h, as shown in Figure S2C. This timeframe ensures complete isopeptide bond formation, enhancing probe avidity as reported in similar self-assembling systems [28]. (The competitive inhibition rate was calculated by (1 − B/B_0_) × 100%, where B_0_ and B are FI_T_/FI_C_ values of negative and positive samples, respectively.)

### 3.6. Optimization of LFIA

The concentration of SpyTagged mimotope, the coating amount of antibody and the dosage of TRFB@SC-STM were optimized by orthogonal experiments, as in Table S2. To balance FI of T-Line and sensitivity, the optimal combination of parameters for the LFIA was as follows: 0.8 mg/mL of UCZ-mAb, 15 μg of SpyTagged mimotope, and 7 μL of TRFB@SC-STM are optimal parameters for the highest ratio of FI_T_ (negative)/(1 − inhibition rate).

**Figure 4:**
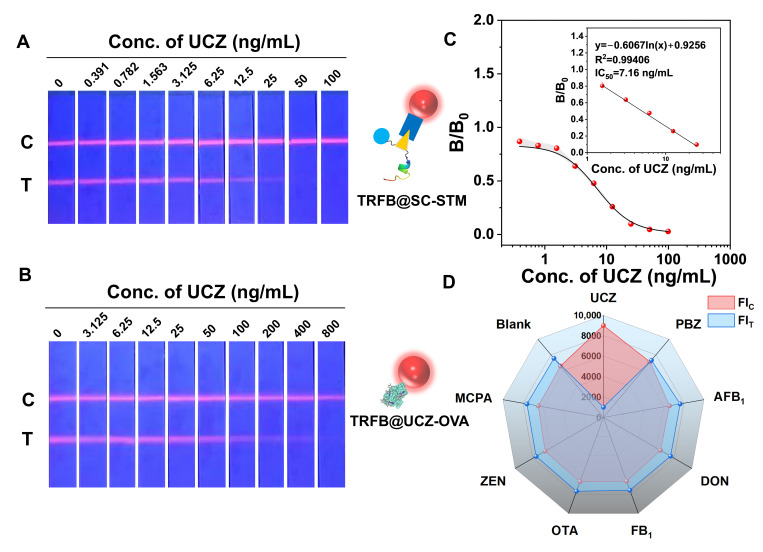
(**A**) Physical drawing of UCZ detection of LFIA based on UCZ SpyTagged mimotope. (**B**) Physical drawing of UCZ detection of LFIA based on UCZ-OVA. (**C**) Calibration curves for UCZ detection of LFIA based on UCZ SpyTagged mimotope. (**D**) Specificity of LFIA based on UCZ SpyTagged mimotope.

The IC_50_ and cut-off of the standard inhibition curve of LFIA are 7.16 ng/mL and 50 ng/mL under the above optimized conditions, shown in Figure 4A,C. Although the IC_50_ of UCZ-OVA and SpyTagged mimotope were similar in the ic-ELISA, the sensitivity of LFIA based on SpyTagged mimotope was about 16-fold higher than that of UCZ-OVA (Figure 4B). The enhanced sensitivity may be attributed to the fact that the natural affinity of mimotope peptides derived from phage display is lower than that of chemically synthesized antigens. Additionally, the antigen–antibody reaction time in LFIA (15 min) is shorter than in ELISA (1 h), and the dissociation rate of mimotope peptides may be faster than that of chemically synthesized antigens.

Figure 4D shows a significant decrease in FI_T_ for UCZ at 25 ng/mL, with negligible variations for other small molecule contaminations at 1 μg/mL, affirming the high specificity of LFIA based on SpyTagged mimotope for the UCZ determination. To meet the requirements of real sample detection, the two parameters, the concentration of methanol and pH value in the buffer, were optimized, and the results are shown in Figure S3A,B. showed that the inhibition has a maximum value at 10% methanol in the buffer, the concentration of methanol and pH value exert profound influence on the detection signal and sensitivity, which may be attributed to the conformational change in the binding site of antibody and antigen at varying concentrations of methanol and pH values.

### 3.7. Optimization of MSIA

According to the principle of the MSIA as shown in Scheme 1C, after the antigen–antibody complex is removed by magnetic separation, the TRFB@SC-STM remaining in the liquid phase system will increase, and the fluorescence signal in the liquid phase will increase. The amplitude of the increase is positively correlated with the concentration of UCZ in the sample, thereby achieving quantitative detection of UCZ.

As shown in Figure S4A, the amount of MB@mAb was optimized, and the S/B rate was highest at the amount of 6.25 μL. To further increase sensitivity, the incubation time in the MSIA was optimized, as shown in Figure S4B; the curve of S/B rate became flat over 15 min. Therefore, 15 min was selected as the optimal incubation time.

**Figure 5:**
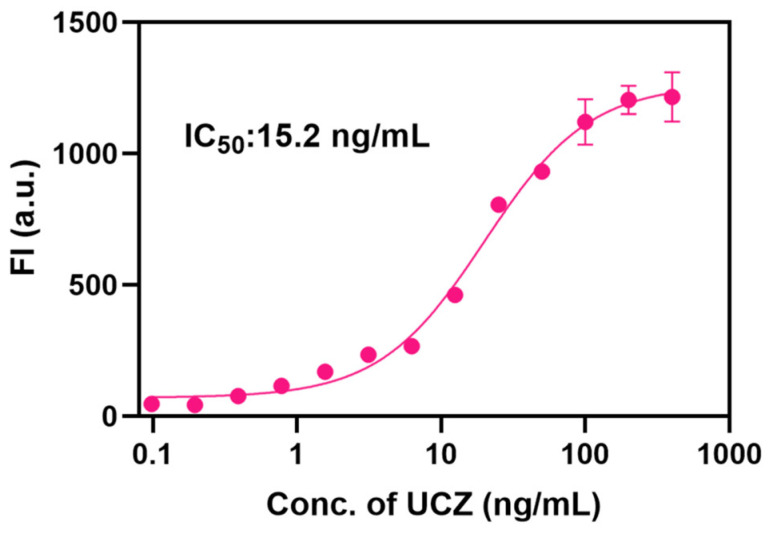
Calibration curves for UCZ detection of MSIA based on UCZ SpyTagged mimotope.

The standard curve for the developed MSIA was measured under these optimized conditions. Figure 5 shows that the IC_50_, LOD (calculated by IC_10_), and linear range based on SpyTagged mimotope can be calculated as 15.2 ng/mL, 2.09 ng/mL, and 4.55–64.58 ng/mL by the standard inhibition curve, indicating that SpyTagged mimotope based on SpyTag/SpyCatcher system is advantageous in MSIA for UCZ.

### 3.8. Matrix Evaluation and Validation Study of LFIA and MSIA Based on SpyTagged Mimotope

**Figure 6:**
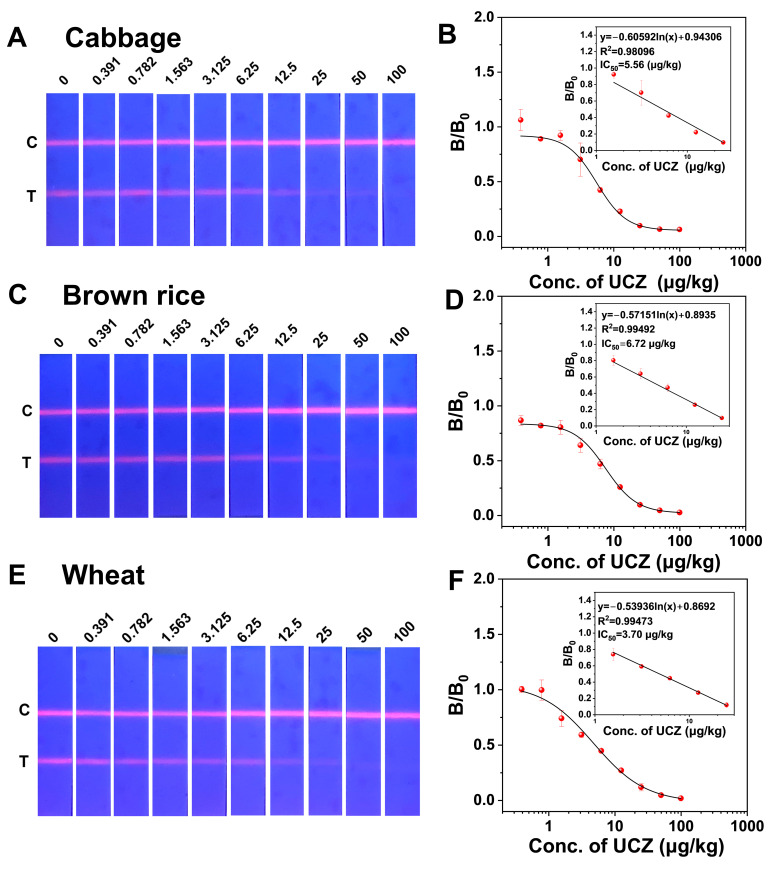
(**A**) Physical drawing of the detection of UCZ in cabbage. (**B**) Calibration curves for detection of UCZ in cabbage. (**C**) Physical drawing of detection of UCZ in brown rice. (**D**) Calibration curves for detection of UCZ in brown rice. (**E**) Physical drawing of detection of UCZ in wheat. (**F**) Calibration curves for detection of UCZ in wheat.

To evaluate the sensitivity and anti-interference ability in a real sample. In this study, cabbage, brown rice, and wheat were selected as representative real matrices. Uniconazole is commonly applied as a plant growth regulator in the cultivation of leafy vegetables and cereal crops, and these commodities are frequently included in routine pesticide residue monitoring programs. Therefore, cabbage (leafy vegetable) and brown rice/wheat (cereal products) not only represent realistic exposure scenarios for UCZ but also cover distinct matrix types for evaluating matrix effects and verifying the practical applicability of the proposed LFIA and MSIA. As shown in Figure 6, the LFIA exhibited linear detection responses for UCZ in cabbage, brown rice, and wheat extracts over the ranges of 2.20–12.81 μg/kg, 3.55–16.02 μg/kg, and 1.36–16.70 μg/kg, respectively. The IC_50_ were 5.56, 6.72, and 3.70 μg/kg, respectively. The cut-off value of the LFIA, as determined by visual assessment, was 50 μg/kg, meeting the requirements for UCZ residue detection. Moreover, the method demonstrated similar performance under different matrix interferences. The LFIA demonstrated high sensitivity in real food samples and, when used in conjunction with a portable fluorescent LFIA reader, has considerable potential for the detection of UCZ in complex food matrices.

**Figure 7:**
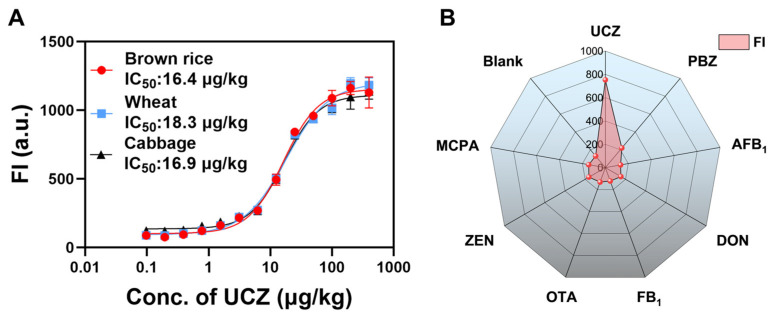
(**A**) Calibration curves for UCZ detection of MSIA in real samples based on UCZ SpyTagged mimotope. (**B**) Specificity.

For the MSIA, the cabbage, brown rice and wheat samples spiked with UCZ were pretreated by QuEChERS for qualitative detection, respectively. (Figure 7A) showed the standard curves in the brown rice, cabbage, and wheat matrix. In brown rice, the IC_50_ was 16.4 μg/kg, the LOD was 2.82 μg/kg with a linear range of 5.43–51.35 μg/kg. In cabbage, the IC_50_ was 16.9 μg/kg, the LOD was 3.39 μg/kg with a linear range of 6.18–48.2 μg/kg. In wheat, the IC_50_ was 18.3 μg/kg, the LOD was 3.72 μg/kg with a linear range of 6.79–53.1 μg/kg. Therefore, our developed method has a better sensitivity for the detection of UCZ in brown rice, cabbage, and wheat. As Figure 7B shows, a significant increase in FI for UCZ at 50 ng/mL, with negligible variations for other small molecule contaminations at 2 μg/mL, affirming the high specificity of MSIA based on SpyTagged mimotope for the UCZ determination.

Additionally, the method complies with the limit requirements of various institutions and can be used to detect UCZ residues in cabbage, brown rice and wheat. Then, we performed quantitative measurements by LFIA, MSIA and LC-MS/MS, as shown in Tables S3 and S4, the recovery for the LFIA ranged from 91.1% to 107.8% with coefficients of variation (CV) lower than 12.3%, for the MSIA ranged from 92.6% to 115.7% with the CVs lower than 9.22% in cabbage, brown rice and wheat. These outcomes validate the assays’ accuracy and precision, with the SpyTagged mimotope enabling eco-friendly, sensitive detection that complements instrumental methods.

## 4. Conclusions

Although the antibody–antigen pair prepared in the laboratory exhibited good performance on the ELISA platform, the LFIA constructed using chemically synthesized antigens showed low sensitivity and failed to meet the detection requirements for real samples. To address this limitation, we obtained a mimotope peptide for UCZ through phage display technology. The mimotope peptide was fused with SpyTag and conjugated by the SpyTag/SpyCatcher system to construct a universal self-assembled fluorescence probe for LFIA, which achieved high sensitivity in line with national standards. Based on the same probes, we developed a wash-free, high-throughput MSIA in rapid quantitative detection for UCZ. The detection time was within 20 min for both LFIA and MSIA based on SpyTagged mimotope.

Furthermore, oriented assembly has effectively addressed the limitations of genetic fusions, enabling the construction of adaptable and multifunctional immunoconjugates. In particular, the employment of SpyTag/SpyCatcher chemistry facilitates the straightforward conjugation of diverse protein or peptide components. These attributes—including oriented assembly, heightened sensitivity, and broad applicability—demonstrate that self-assembly strategies provide a powerful approach for advancing immunoassay systems.

## Data Availability

The original contributions presented in this study are included in the article/Supplementary Material. Further inquiries can be directed to the corresponding author.

## Supplementary Materials

The following supporting information can be downloaded at: https://www.mdpi.com/article/10.3390/foods14244358/s1, Figure S1. Characterization of TRFB@SC-STM. (A) DH of TRFB, TRFB@SC, and TRFB@SC-STM. (B) Zeta potentials of TRFB-free, TRFB-SpyTag, and TRFB-mimotope. (C) TEM images of TRFBs. (D) Excitation and emission spectra of TRFB@SC-STM. Figure S2. Parameter optimization of TRFB probes. (A) pH value. (B)The volume of SpyCatcher. (C)The incubation time of UCZ SpyTagged mimotope and TRFB@SC. Figure S3. Optimization of LFIA buffer. (A) The concentration of methanol. (B) pH value. Figure S4. Optimization of MSIA. (A) The volume of MB@mAb. (B) Incubation. Table S1. The biopanning strategy for phage displayed mimotope peptide. Table S2. Orthogonal test for parameter optimization of LFIA. Table S3. The precision and accuracy of LFIA. Table S4. The precision and accuracy of MSIA. Table S5. The amino acid sequence of UCZ SpyTagged mimotope, UCZ-mAb, and SpyCatcher. Table S6. Comparison of UCZ detection methods based on immunoassay.
