# Supplementary material for: SpyTagged Mimotope Peptide Mediated Competitive Antigen-Based Rapid Quantitative Immunoassays for Uniconazole Residue
Source: Foods. 2025 Dec 18;14(24):4358. doi: 10.3390/foods14244358 (PMC12733066; doi:10.3390/foods14244358)
Supplement: Supplementary file 1 [file foods-14-04358-s001.zip › foods-3964741-supplementary.pdf]

# SpyTagged Mimotope Peptide Mediated Competitive Antigen-Based Rapid Quantitative Immunoassays for Uniconazole Residue

**Tailong Wei [1], Xiao Chen [1], Chong Cai [1], Yuanzhen Guo [1], Mengjun Zhou [2], Qiannan Gao [1] and Qinghua He [1,3,4,*]**

[1] State Key Laboratory of Food Science and Resources, Nanchang University, No. 235 Nanjing East Road, Nanchang 330047, China; 357900210015@email.ncu.edu.cn (T.W.); 19326409190@163.com (X.C.); 357900240012@email.ncu.edu.cn (C.C.); 407900230007@email.ncu.edu.cn (Y.G.); 417900230134@email.ncu.edu.cn (Q.G.)

[2] Jiangxi General Institute of Testing and Certification Instituto for Food Control, Nanchang 330052, China; 13767176735@163.com

[3] Sino-German Joint Research Institute, Nanchang University, No. 235 Nanjing East Road, Nanchang 330047, China

[4] In Vitro Diagnostic Technology Innovation Center for Nanobody, No. 1166 Yiyuan Road, Nanchang 330038, China

* Correspondence: heqinghua@ncu.edu.cn

## Figure captions:

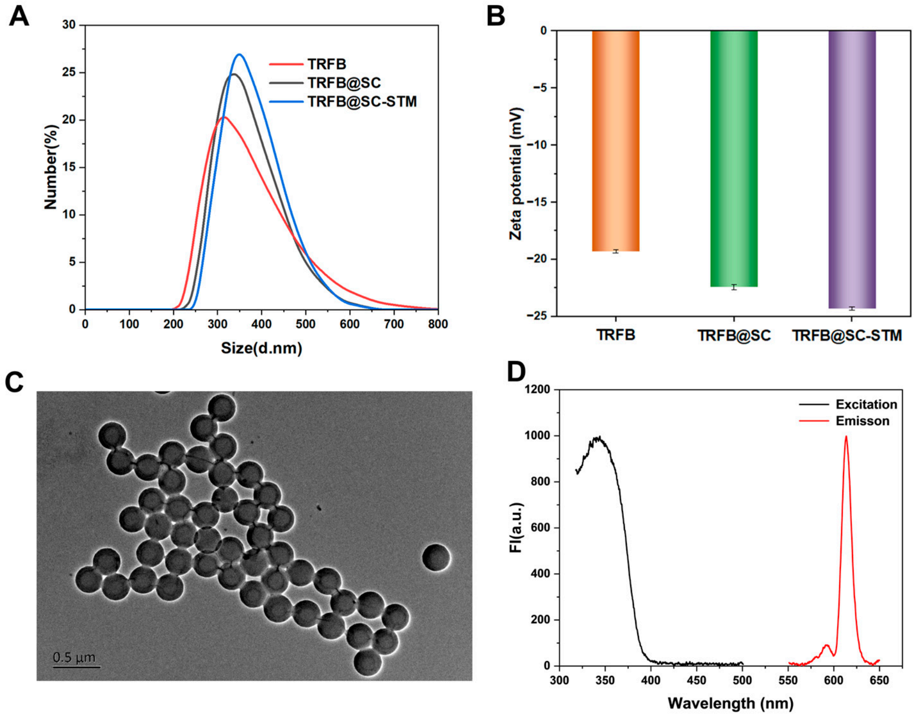


**Figure S1.** Characterization of TRFB@SC-STM. (**A**) $D_H$ of TRFB, TRFB@SC, and TRFB@SC-STM. (**B**) Zeta potentials of TRFB-free, TRFB-SpyTag, and TRFB-mimotope. (**C**) TEM images of TRFBs. (**D**) Excitation and emission spectra of TRFB@SC-STM.

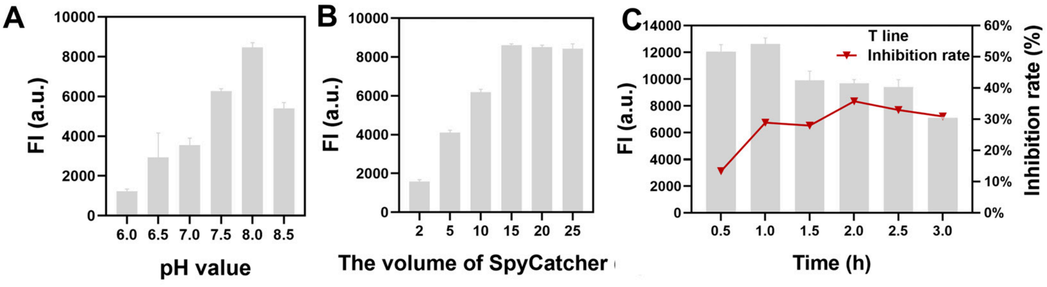


**Figure S2.** Parameter optimization of TRFB probes. (**A**) pH value. (**B**) The volume of SpyCatcher. (**C**) The incubation time of UCZ SpyTagged mimotope and TRFB@SC.

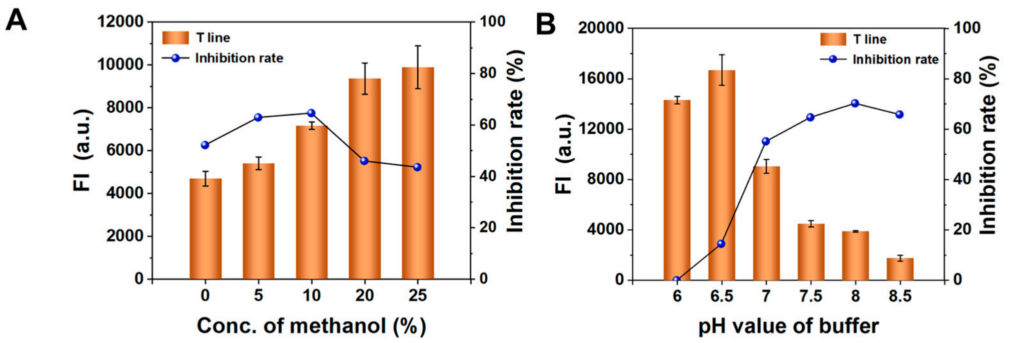


**Figure S3.** Optimization of LFIA buffer. (**A**) The concentration of methanol. (**B**) pH value.

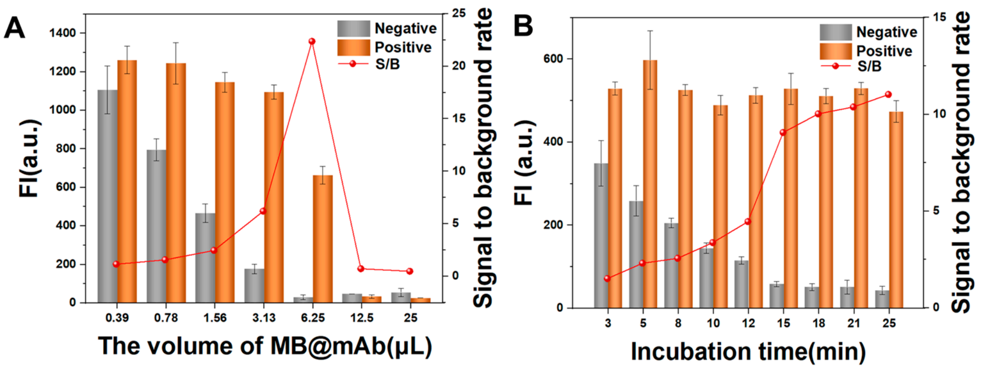


**Figure S4.** Optimization of MSIA. (**A**) The volume of MB@mAb. (**B**) Incubation.

**Table S1.** The biopanning strategy for phage displayed mimotope peptide.

| Round | UCZ-mAb (μg/mL) | Input (pfu) | Blocking Reagent | PBST | Time (min) |
|---|---|---|---|---|---|
| 1 | 100 | $2x10^{11}$ | 3% BSA | 0.1% | 15 |
| 2 | 50 | $2x10^{11}$ | 3% OVA | 0.25% | 12 |
| 3 | 30 | $2x10^{11}$ | 3% BSA | 0.5% | 8 |

**Table S2.** Orthogonal test for parameter optimization of LFIA.

| NO. | UCZ-mAb (mg/mL) | SpyTag mimotope (μg) | TRFB@SC-STM (μL) | $FI_T$ (negative) | $FI_T$ (positive) | Inhibition rate (%)[a] |
|---|---|---|---|---|---|---|
| 1 | 0.5 | 10 | 3 | 2983 | 1509 | 52.67 |
| 2 | 0.5 | 15 | 5 | 5575 | 2299 | 64.77 |
| 3 | 0.5 | 20 | 7 | 8326 | 4608 | 52.11 |

| | | | | | | |
|---|---|---|---|---|---|---|
| 4 | 0.8 | 10 | 5 | 4737 | 3092 | 46.11 |
| 5 | 0.8 | 15 | 7 | 8349 | 4428 | 55.89 |
| 6 | 0.8 | 20 | 3 | 5787 | 3475 | 50.62 |
| 7 | 1.2 | 10 | 7 | 9347 | 5697 | 48.44 |
| 8 | 1.2 | 15 | 3 | 5241 | 3488 | 48.77 |
| 9 | 1.2 | 20 | 5 | 8395 | 3229 | 55.40 |

Note: The concentration of UCZ in positive sample is 10 ng/mL.

**Table S3.** The precision and accuracy of LFIA.

| Spiked UCZ (μg/kg) | Cabbage | | | Brown rice | | | Wheat | | |
|---|---|---|---|---|---|---|---|---|---|
| | Mean (μg/kg) | Rcovery rate(%) | CV (%) | Mean (μg/kg) | Rcovery rate(%) | CV(%) | Mean (μg/kg) | Rcovery rate(%) | CV(%) |
| 5 | 4.83 | 96.53 | 10.21 | 4.62 | 92.4 | 9.76 | 4.56 | 91.13 | 10.21 |
| 10 | 9.61 | 96.06 | 7.64 | 9.69 | 96.93 | 8.43 | 10.43 | 104.3 | 12.34 |
| 20 | 20.56 | 102.82 | 6.73 | 20.94 | 104.71 | 7.32 | 21.56 | 107.81 | 8.48 |

**Table S4.** The precision and accuracy of MSIA.

| Sample | Spiked (μg/kg) | MSIA | | | LC-MS/MS | | |
|---|---|---|---|---|---|---|---|
| | | Detection value | Recovery (%) | CV (%) | Detection value | Recovery (%) | CV (%) |
| | | Mean (μg/kg) | | | Mean (μg/kg) | | |
| Cabbage | 20 | 18.84 | 94.2 | 3.46 | 18.23 | 91.2 | 3.32 |
| | 50 | 48.32 | 96.6 | 4.67 | 49.58 | 99.2 | 5.31 |
| | 100 | 105.30 | 105.3 | 8.61 | 104.51 | 104.5 | 3.46 |
| Brown rice | 20 | 23.14 | 115.7 | 7.41 | 18.32 | 91.6 | 4.14 |
| | 50 | 52.11 | 104.2 | 6.34 | 48.83 | 97.7 | 3.94 |
| | 100 | 103 | 103.4 | 7.45 | 106.17 | 106.2 | 5.21 |
| Wheat | 20 | 19.41 | 96.1 | 8.31 | 21.34 | 106.7 | 3.74 |
| | 50 | 46.32 | 92.6 | 9.22 | 47.35 | 94.7 | 3.86 |
| | 100 | 94.35 | 94.4 | 7.64 | 93.62 | 93.6 | 4.24 |

**Table S5.** The amino acid sequence of UCZ SpyTagged mimotope, UCZ-mAb, and **SpyCatcher.**

Amino acid sequence of STMP. TrxA (cyan), SpyTag (orange), UCZ mimotope (green).

| | |
|---|---|
| **UCZ SpyTagged mimotope** | MSDKIIHLTDDSFDTDVLKADGAILVDFWAEWCGPCKMIAPILDEIADEYQGKLTVAKLNIDQNPG<br>TAPKYGIRGIPTLLLFKNGEVAATKVGALSKGQLKEFLDANLAGSGSGHMHHHHHHSSGLVPRGS<br>GMKETAAAKFERQHMDSPDLGTDDDDKAMADIGSAHIVMVDAYKPTKGGGSGGGSGGGSIPFKE<br>GLGFISGGGGSGGGSGGGSLEHHHHHH* |
| **UCZ mAb** | V QVQLQQPGAELVRPGASVKLSCKASGYTFTVYYIYWVKQRPGQGLEWIGGIHPRNGGSYYNA<br>H KFRNRATLTVDKSSNTAYMQFSSLTSEDSAVYYCTRGIYYDGKFGERAMDYWGQGTSVTVSS<br>V DIVMTQTPNTLSVTIGQPASISCKSSQSLLYSDGKTYLHWLLQSPGQSPKLLIYLVSKLESGVPDRF<br>L SGSGSGTDFTLKISRVEAEDLGVYYCVQAAHLPHTFGSGTKLEIK |
| **SpyCatcher** | MVDTLSGLSSEQGQSGDMTIEEDSATHIKFSKRDEDGKELAGATMELRDSSGKTISTWISDGQVKDF<br>YLYPGKYTFVETAAPDGYEVATAITFTVNEQGQVTVNGKATKGDAHIGGGGSGGGGSLEHHHHH<br>H* |

**Table S6.** Comparison of UCZ detection methods based on immunoassay.

| **Platform** | **Antigen** | **Antibody** | **Signal Materials** | **LOD in Buffer (ng/mL)** | **LOD in Matrix (μg/kg)** | **Detection Time (min)** | **Detection Sample** | **Ref.** |
|---|---|---|---|---|---|---|---|---|
| ELISA | UCZ-OVA | pAb | HRP | 1.82 | - | - | Water , soil | [1] |
| ELISA | UCZ-HRP | pAb | HRP | 0.022 | - | 75 | Apple | [2] |
| Electrochemical Immunosensor | - | mAb | - | 8 | - | 90 | Cabbage | [3] |
| ICA | Mimotope peptide | mAb | TRF | 50(vLod) | 50(vLod) | 15 | Cabbage, wheat, brown rice | This work |
| MSIA | Mimotope peptide | mAb | TRF | 2.09 | 2.82-3.73 | 20 | Cabbage, wheat, brown rice | This work |

## References


1. Jiang X.; Zhang Z.; Wu N.; Shi H.; Wang M. Development of an Enzyme-Linked Immunosorbent Assay for Detection of Uniconazole. *J. Anal. Sci.* **2010**, *26*, 677–680. Available online: http://en.cnki.com.cn/Article_en/CJFDTOTAL-FXKX201006017.htm(accessed on 15 November 2025.).
2. Zhang L.; Wang Q.; Wang N.; Liu S. Determination of Uniconazole Residue in Apple by Direct Competitive Enzyme-Linked Immunosorbent Assay and High Performance Liquid Chromatography. *Chin. J. Anal. Chem.* **2012**, *40*, 1730–1734, doi:10.3724/SP.J.1096.2012.20123.
3. Wei L.; Liu L.; Lu H.; Teng Z.; Kang H.; Wang C.; Hu X. An Impedance Immunosensor for Detection of Uniconazole Based on Screen Printed Electrode. *Chin. J. Anal. Chem.* **2016**, *44*, 258–264, doi:10.11895/j.issn.0253-3820.150391.
